# Identification of potential biomarkers associated with mitochondrial oxidative stress in idiopathic pulmonary arterial hypertension via bioinformatic and experimental analysis

**DOI:** 10.1038/s41598-025-31150-7

**Published:** 2025-12-09

**Authors:** Yanting Zhu, Xiaoming Wang, Xiaohui Yan, Yan Sun, Qiong Wang, Cui Zhai

**Affiliations:** 1 Department of Nephropathy, Shaanxi Provincial People’s Hospital, Xi’an, 710068 Shaanxi People’s Republic of China; 2 Institute of Basic and Translational Medicine, Xi’an Medical University, Xi’an, 710021 Shaanxi People’s Republic of China

**Keywords:** Pulmonary arterial hypertension, Mitochondrial oxidative stress, Potential biomarkers, Bioinformatics, Chronic kidney disease, Diagnostic markers

## Abstract

Idiopathic pulmonary arterial hypertension (IPAH) is a progressive and devastating disorder. Mitochondrial oxidative stress has been found to be involved in PAH development. This study aims to explore potential mitochondrial oxidative stress-related biomarkers in IPAH via integrated bioinformatics, validate these genes in MCT-induced rat PAH model and examine the functional roles of COX6B1 and HMGCL in mitochondrial oxidative stress in PDGF-induced pulmonary arterial smooth muscle cells (PASMCs). GSE15197 was downloaded from GEO database. Genes associated with mitochondria and oxidative stress were obtained from Molecular Signatures Database and MitoCarta3.0 databases. Weighted gene co-expression network analysis (WGCNA) was used. GO and KEGG enrichment analysis were utilized. Furthermore, machine learning algorithms (LASSO, SVM-RFE and RF) were constructed to screen hub genes. A nomogram model based on the hub genes were performed for evaluation the diagnostic value, and the expression of hub genes were validated by real-time quantitative PCR and Western blot in MCT-induced rat PAH model. Finally, COX6B1 and HMGCL specific siRNAs were transfected into PDGF-induced PASMCs, mitochondrial ROS (mtROS) level and MnSOD activity were examined. A total of 18 genes related to mitochondrial oxidative stress were identified in IPAH. Two hub genes (COX6B1 and HMGCL) were finally determined as potential biomarkers. Based on the 2 genes, a nomogram model was constructed. This model exhibited remarkable diagnostic efficiency values for IPAH. The expressions of COX6B1 and HMGCL were found to be upregulated in MCT-induced PAH model, in agreement with the findings of bioinformatics analysis. While, transfection with either COX6B1 siRNA or HMGCL siRNA suppressed the abnormal accumulation of mtROS and restored MnSOD activity in PDGF-induced PASMCs. Our results indicated that two mitochondrial oxidative stress-related genes (COX6B1 and HMGCL) were identified as diagnostic biomarkers and may serve as potential targets for IPAH development, although further research is necessary.

**Supplementary Information:**

The online version contains supplementary material available at 10.1038/s41598-025-31150-7.

## Introduction

Pulmonary arterial hypertension (PAH) is a progressive disease characterized by increased pulmonary vascular resistance and irreversible vascular remodeling, which involves various factors and severely impairs cardiovascular and respiratory function, eventually leading to right heart failure, even death^1^. Due to a high mortality rate and limited treatment options, PAH has become a global health issue that urgently needs to be addressed. PAH is divided into five groups and idiopathic pulmonary arterial hypertension (IPAH) is the most common subtype of PAH occurred in the absence of etiology. The pathogenesis of IPAH is complex, in which pulmonary arterial smooth muscle cells (PASMCs) homeostasis imbalance playing a critical role.

Mitochondria, the energy factories of cells, is not only the primary sites of ATP generation but also a major source of reactive oxygen species (ROS)^2^. Mitochondrial ROS (mtROS) is primarily released from the mitochondrial electron transport chain. Mitochondria also possess a sophisticated ROS scavenging network to regulate excessive mtROS generation^3^. Under physiological conditions, mtROS participates in signal transduction, vascular tone regulation, and oxygen radical sensing^4^. However, excessive ROS damages mitochondrial lipids, nucleic acids and proteins, leading to mitochondrial oxidative stress (MOS), which triggers mitochondrial dysfunction and cellular homeostasis imbalance^6^. Previous studies have shown that an imbalance between mtROS production and clearance is commonly observed in PASMCs, which triggers increased cytosolic calcium levels, apoptosis resistance and abnormal proliferation, resulting in the occurrence of PAH^6^. Therefore, further research on mechanisms and biomarkers targeting mitochondrial oxidative stress is needed to enable early diagnosis and more effective treatments of IPAH.

This study aims to explore hub genes that are differentially expressed and associated with mitochondrial oxidative stress in IPAH using bioinformatics methods to examine chip data sourced from the GEO database. We further verify the expressions of the hub genes in rat PAH model and examine the functional roles of these genes in mitochondrial oxidative stress in PDGF-induced PASMCs, which provided possible indicators and treatment targets for IPAH.

## Results

### Identification of candidate mitochondrial oxidative stress-related genes in IPAH

**Fig. 1:**
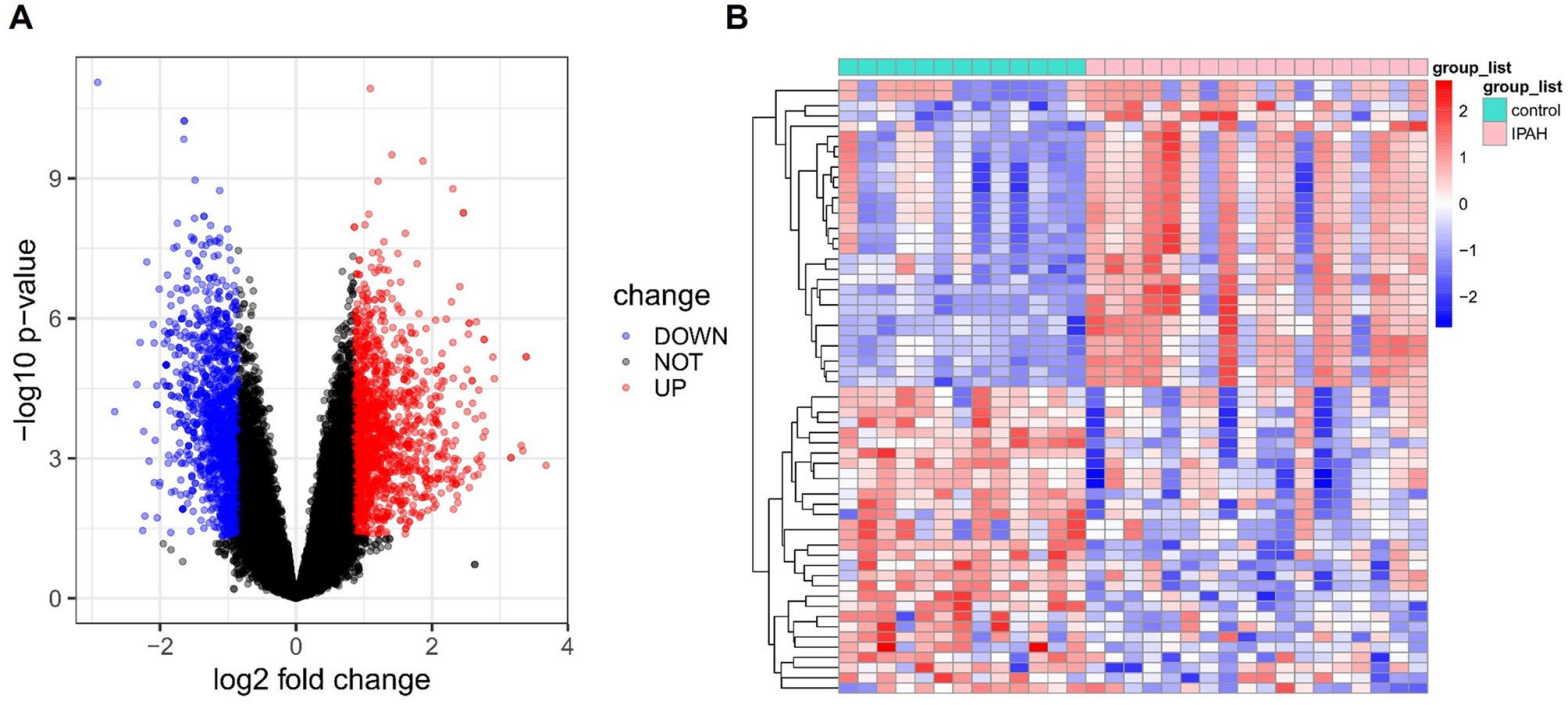
Identification of DEGs in IPAH. (**A**) Volcano plots of DEGs between IPAH and control groups. (**B**) Heatmap of the top 30 upregulated and top 30 downregulated DEGs.

The GSE15197 dataset was selected and DEGs between IPAH patients and normal controls were analyzed for the present study. In total, there were 3043 DEGs identified between IPAH and controls, of which 1594 were upregulated and 1449 were downregulated. The volcano plots of the DEGs are shown in Fig. 1A. The top 30 upregulated DEGs and top 30 downregulated DEGs are shown in the heatmap (Fig. 1B).

**Fig. 2:**
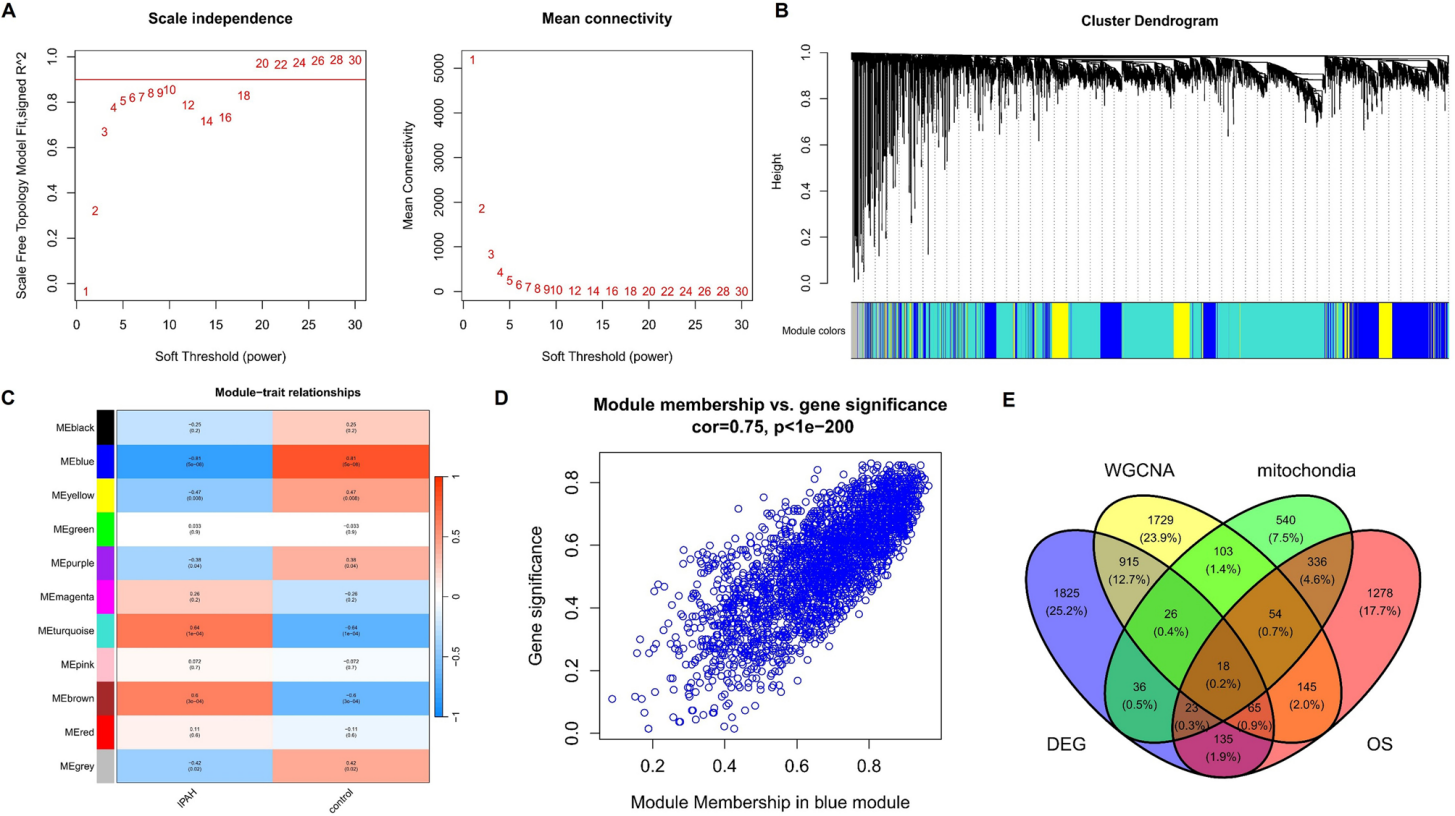
WGCNA in IPAH and candidate mitochondrial oxidative stress-related genes identification. (**A**) Network topology analysis for selecting the soft-thresholding power. (**B**) Gene cluster dendrogram and module assignment. (**C**) Heatmap of module-trait relationships. (**D**) Scatterplot displaying the correlation between module membership in the blue module and gene significance for IPAH. (**E**) Venn diagram illustrating the overlap between DEGs, blue module, mitochondria-related genes and oxidative stress-related genes.

In the WGCNA analysis, with the soft-threshold power established at 10, the network reached the desired scale-free topology with a model fit index of 0.9 (Fig. 2A), and then eleven modules were identified (Fig. 2B and C). The blue module was chosen for further investigation based on the most robust positive correlation with IPAH (Fig. 2D).

There were 18 candidate genes related to IPAH, mitochondria and oxidative stress by intersecting 3043 DEGs, 3055 modular genes, 1136 mitochondrial genes and 2054 oxidative stress-associated genes (Fig. 2E).

### Function analysis of candidate mitochondrial oxidative stress-related genes

**Fig. 3:**
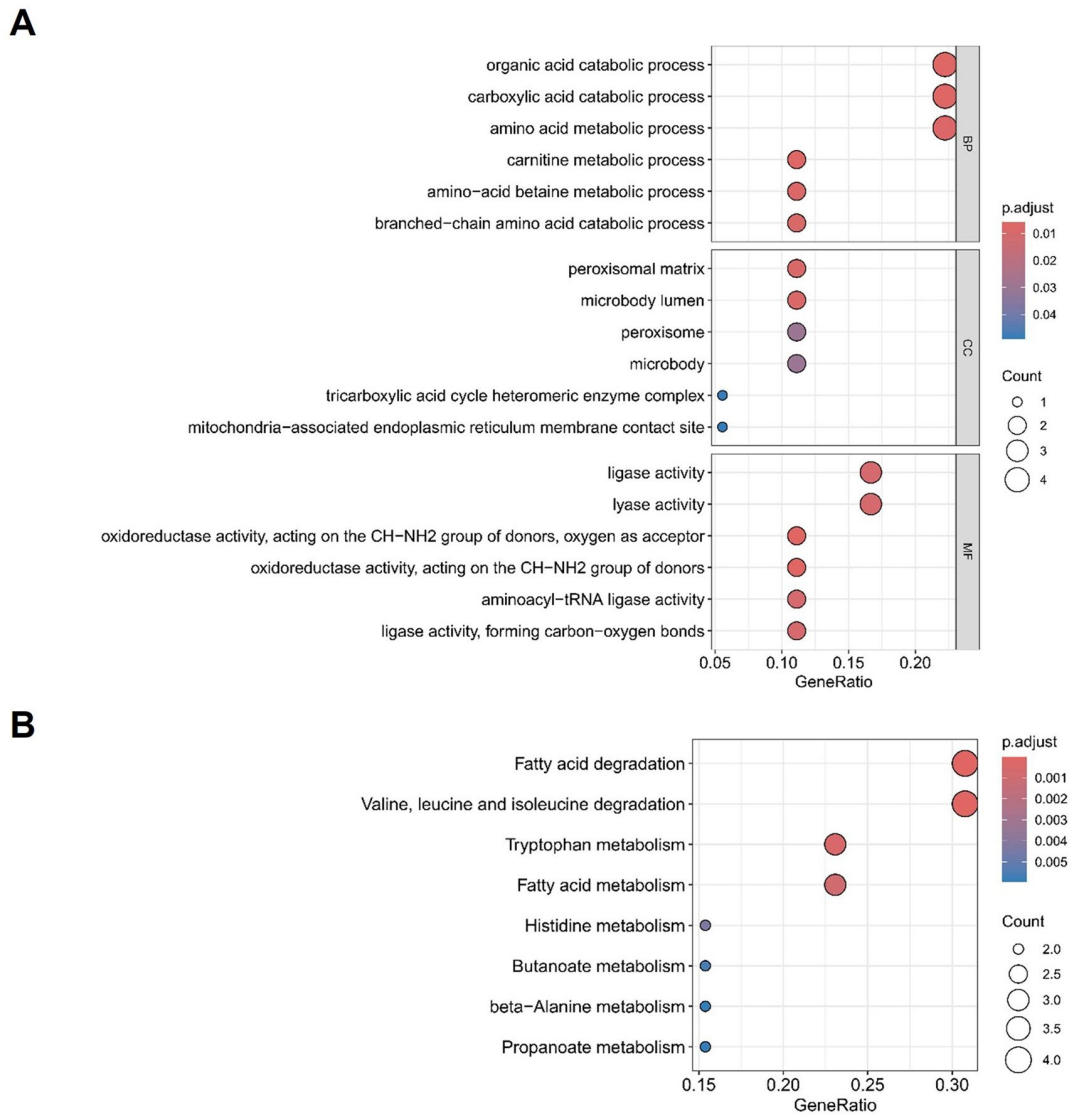
GO and KEGG enrichment analysis of 18 candidate mitochondrial oxidative stress-related genes. (**A**) Bubble plots of GO enrichment analysis. (**B**) KEGG enrichment analysis. The figure was generated based on data from the KEGG pathway database^24–26^.

GO enrichment analysis and KEGG pathway analysis performed for the candidate mitochondrial oxidative stress-related genes are shown in Fig. 3. BP terms showed that the genes were enriched in organic acid catabolic process, carboxylic acid catabolic process, amino acid metabolic process, carnitine metabolic process, amino-acid betaine metabolic process and branched-chain amino acid catabolic process. In CC terms, mitochondrial oxidative stress-related genes mainly participated in peroxisomal matrix, microbody lumen, peroxisome, microbody, tricarboxylic acid cycle heteromeric enzyme complex and mitochondria-associated endoplasmic reticulum membrane contact site. Ligase activity, lyase activity, oxidoreductase activity, acting on the CH-NH2 group of donors, oxygen as acceptor, aminoacyl-tRNA ligase activity, ligase activity, forming carbon-oxygen bonds were most obviously enriched for mitochondrial oxidative stress-related genes in the MF category. The KEGG enrichment analysis was primarily involved in fatty acid degradation, valine, leucine and isoleucine degradation, tryptophan metabolism, fatty acid metabolism, histidine metabolism, butanoate metabolism, beta-Alanine metabolism, propanoate metabolism and so on.

### Identification of hub genes related to mitochondrial oxidative stress

**Fig. 4:**
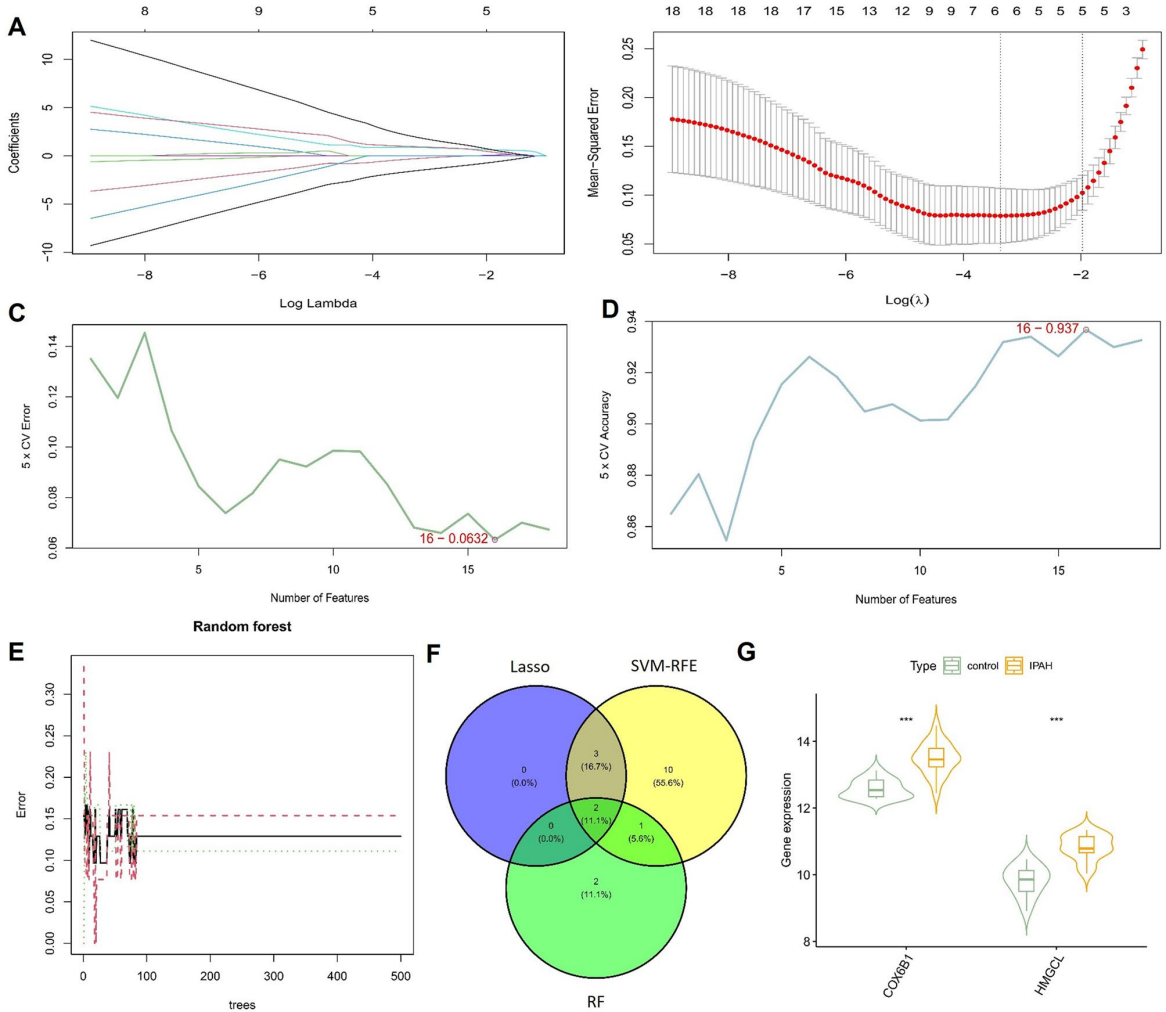
Identification of hub genes based on machine learning. (**A**–**B**) Coefficient paths at different λ values (**A**) and ten-fold cross-validation curve (**B**) in the LASSO regression. (**C**–**D**) The error rate (**C**) and accuracy rate (**D**) of the number of variables in the SVM-RFE method. (**E**) The performance of the RF model varies with the number of trees. (**F**) Venn diagram illustrating the hub genes selected by the three algorithms. (**G**) Relative expression of two hub genes (COX6B1 and HMGCL) between IPAH and control groups. ∗∗∗*P* < 0.001 versus control group.

The LASSO logistic regression algorithm was employed using gene expression levels from the candidate mitochondrial oxidative stress-related genes as features to screen for hub genes. 5 genes from the candidate mitochondrial oxidative stress-related genes were identified as key characteristic genes (Fig. 4A, B). In addition, the candidate mitochondrial oxidative stress-related genes were analyzed using the SVM-RFE machine learning algorithm. As depicted in Fig. 4C, D, the SVM model had the maximum classification accuracy and the minimum classification error. Thus, 16 genes were extracted from the model as key characteristic genes. The RF algorithm was employed to scrutinize the 18 candidate mitochondrial oxidative stress-related genes for potential biomarkers of IPAH, revealing 5 genes of potential diagnostic relevance with importance up to 1.5 (Fig. 4E). A Venn plot was constructed to reveal 2 hub genes coselected by all machine learning methods, comprising COX6B1 and HMGCL (Fig. 4F). Violin plots revealed significant differences in the expression levels of COX6B1 and HMGCL between IPAH and control (Fig. 4G).

### Establishment of a diagnostic model of IPAH samples based on hub genes

**Fig. 5:**
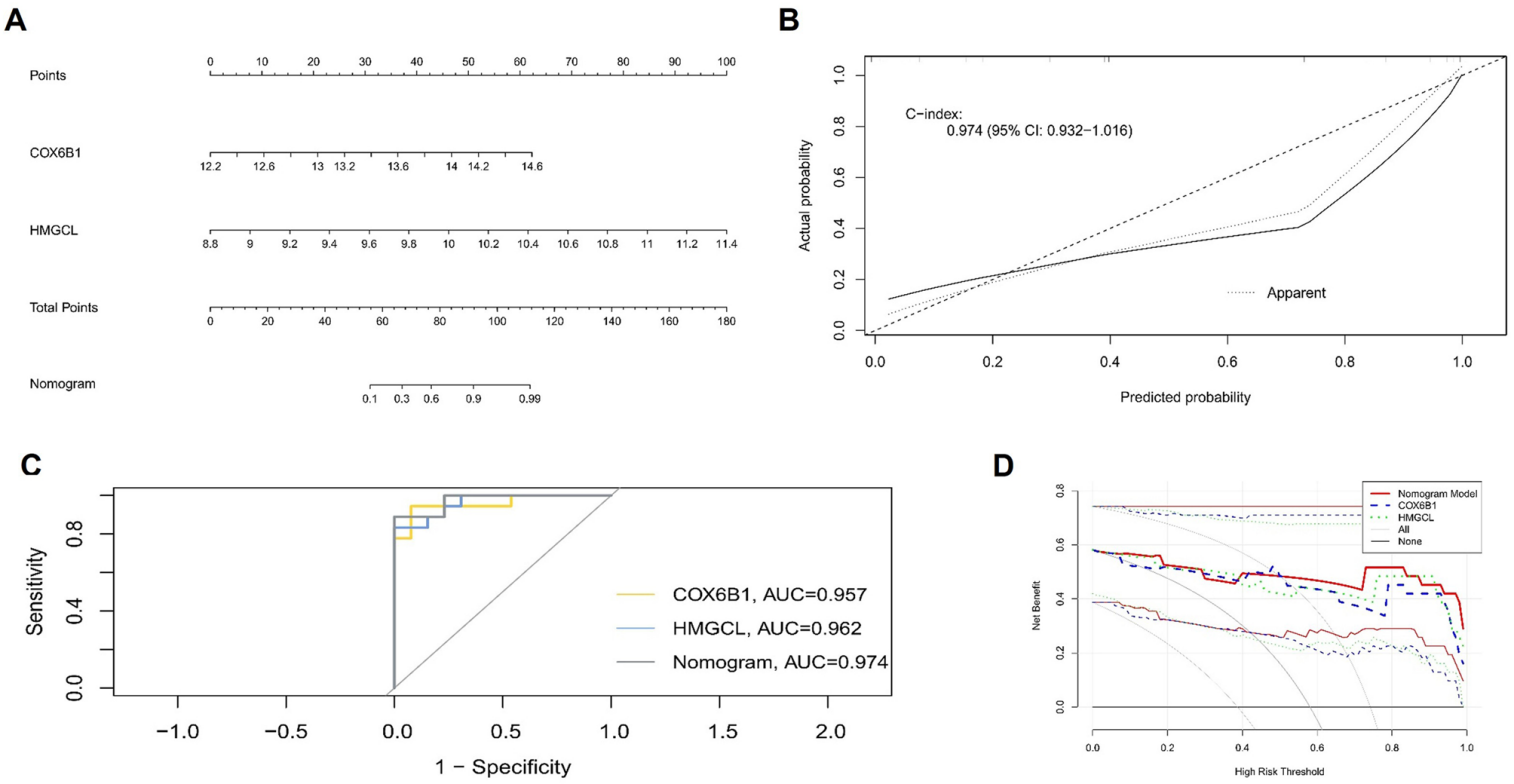
Development of the nomogram model and efficacy assessment. (**A**) Nomogram model for predicting IPAH risk based on the expression levels of COX6B1 and HMGCL. (**B**) Calibration curve of the nomogram model. (**C**) ROC curves evaluating the predictive performance of individual genes and nomogram model. (**D**) DCA for the nomogram model.

A nomogram model was established to evaluated the weights and disease risk predictive power of the 2 hub genes (Fig. 5A). The calibration curve confirmed that the bias-corrected model had high prediction accuracy on an independent dataset (Fig. 5B). Furthermore, a combined ROC analysis of the 2 hub genes showed an AUC was 0.974, extremely high diagnostic efficiency (Fig. 5C). Decision curve analysis (DCA) confirmed the performance of this model (Fig. 5D).

### Validation of hub genes expressions in rat PAH model

**Fig. 6:**
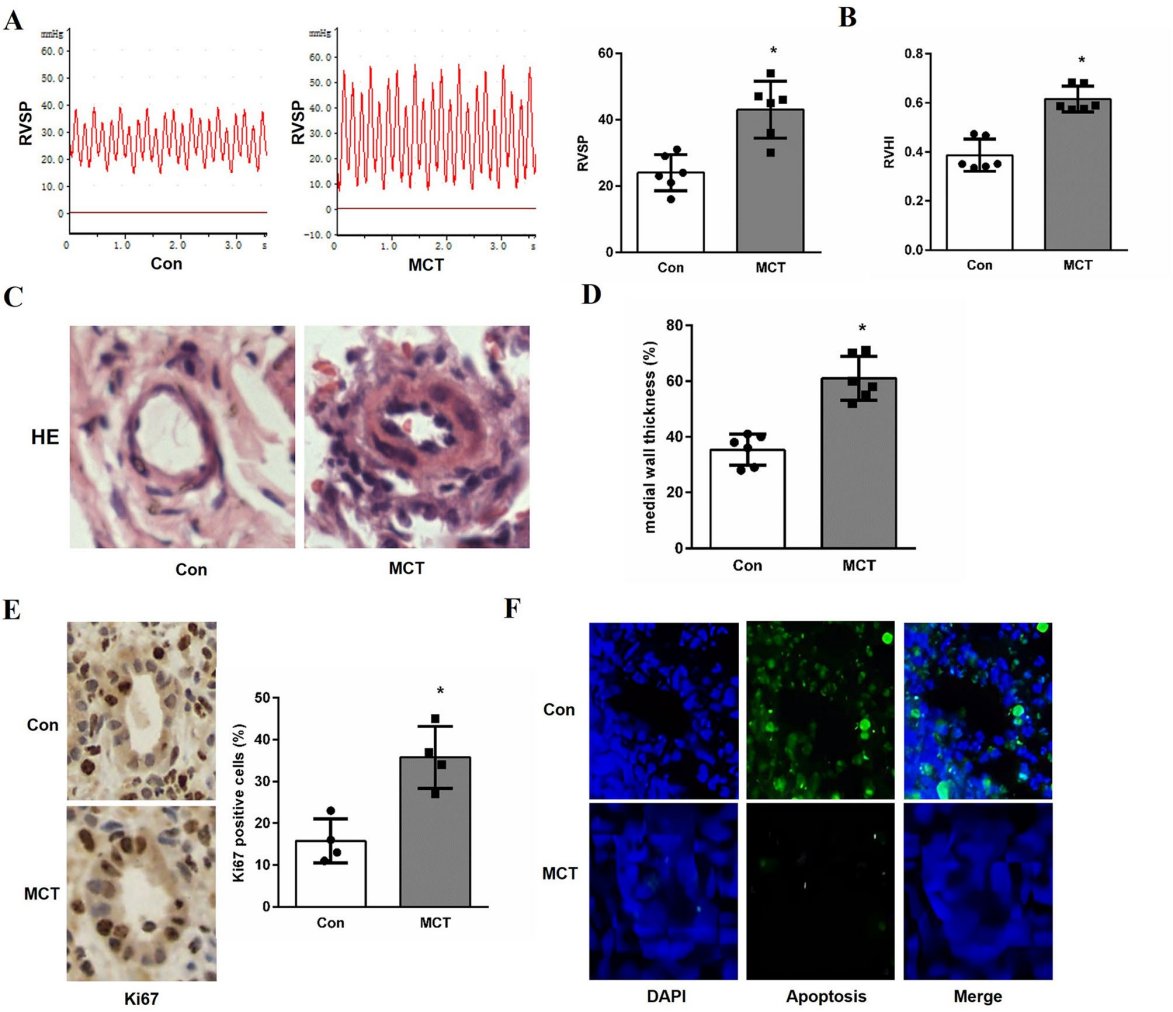
PAH progression and physiological assessment in MCT-induced rat model. (**A**) Representative image of RVSP measurement (*n* = 6 per group). *P < 0.05 versus control group. (**B**) Changes of RVHI in each group (*n* = 6 per group). *P < 0.05 versus control group. (**C**) Representative HE-staining of small pulmonary vessels. (**D**) Quantitative analysis of the wall thickness of pulmonary artery in different groups (*n* = 6 per group). *P < 0.05 versus control group. (**E**) Ki67 immunohistochemistry staining in the arterial medial layer (*n* = 4 per group) (×400). *P < 0.05 versus control group. (**F**) Representative image of TUNEL staining.

As shown in Fig. 6A, RVSP measured after 4 weeks in MCT-induced rats was significantly elevated compared with control rats. Another parameter RVHI exhibited the similar results. The RVHI in MCT-treated rats was dramatically higher than that in the control rats (Fig. 6B). Remodeling of the pulmonary artery was then examined by histological analysis. The MCT-treated rats showed a notable vascular remodeling and increase in medial wall thickness in comparison to the control rats (Fig. 6C, D). Furthermore, cells proliferation and apoptosis, the major pathological changes of pulmonary arterial remodeling in PAH, was examined using Ki67 and TUNEL staining. As depicted in Fig. 6E, the percentage of Ki67 positive cells was markedly increased in MCT-induced rats compared to the control rats. A reduction in the percentage of apoptotic cells was observed in the MCT group compared to the control group (Fig. 6F). These results indicate that the PAH model was successful established with the pulmonary arterial remodeling by increased proliferation and reduced apoptosis of cells.

**Fig. 7:**
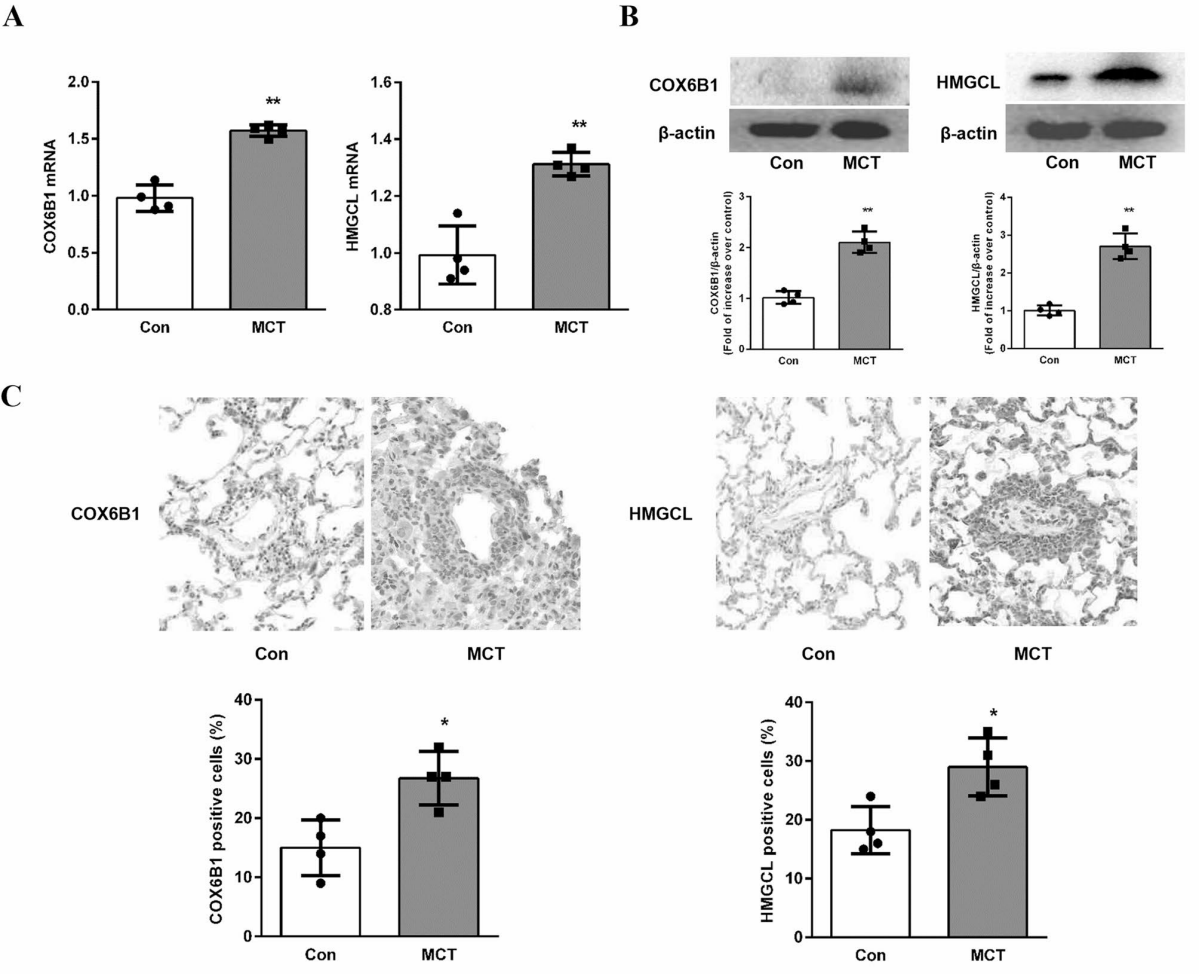
Validation of hub genes expressions in MCT-induced PAH model. (**A**) mRNA expressions of COX6B1 and HMGCL in lung tissue by RT-qPCR (*n* = 4 per group). **P < 0.01 versus control group. (**B**) Western blot of COX6B1 and HMGCL in lung tissues. β-actin served as loading control. A representative blot and quantification of bands are shown (*n* = 4 per group). **P < 0.01 versus control group. (**C**) Immunohistochemistry staining of COX6B1 and HMGCL in vasculature of lung tissues (*n* = 4 per group). *P < 0.05 versus control group.

Furthermore, the mRNA and protein expressions of the 2 hub genes were analyzed in lung tissues using RT-qPCR and western blot, respectively. As shown in Fig. 7A, the mRNA expression levels of COX6B1 and HMGCL in MCT-induced rats were significantly upregulated compared with controls, aligned with the findings of bioinformatics analysis. Similar results were also observed in protein expressions of COX6B1 and HMGCL (Fig. 7B). The result was also confirmed by immunohistochemistry in which COX6B1 and HMGCL were predominantly expressed in the medial layer of vasculature (Fig. 7C).

### Knockdown of hub genes inhibits mitochondrial oxidative stress in PDGF-induced PASMCs

**Fig. 8:**
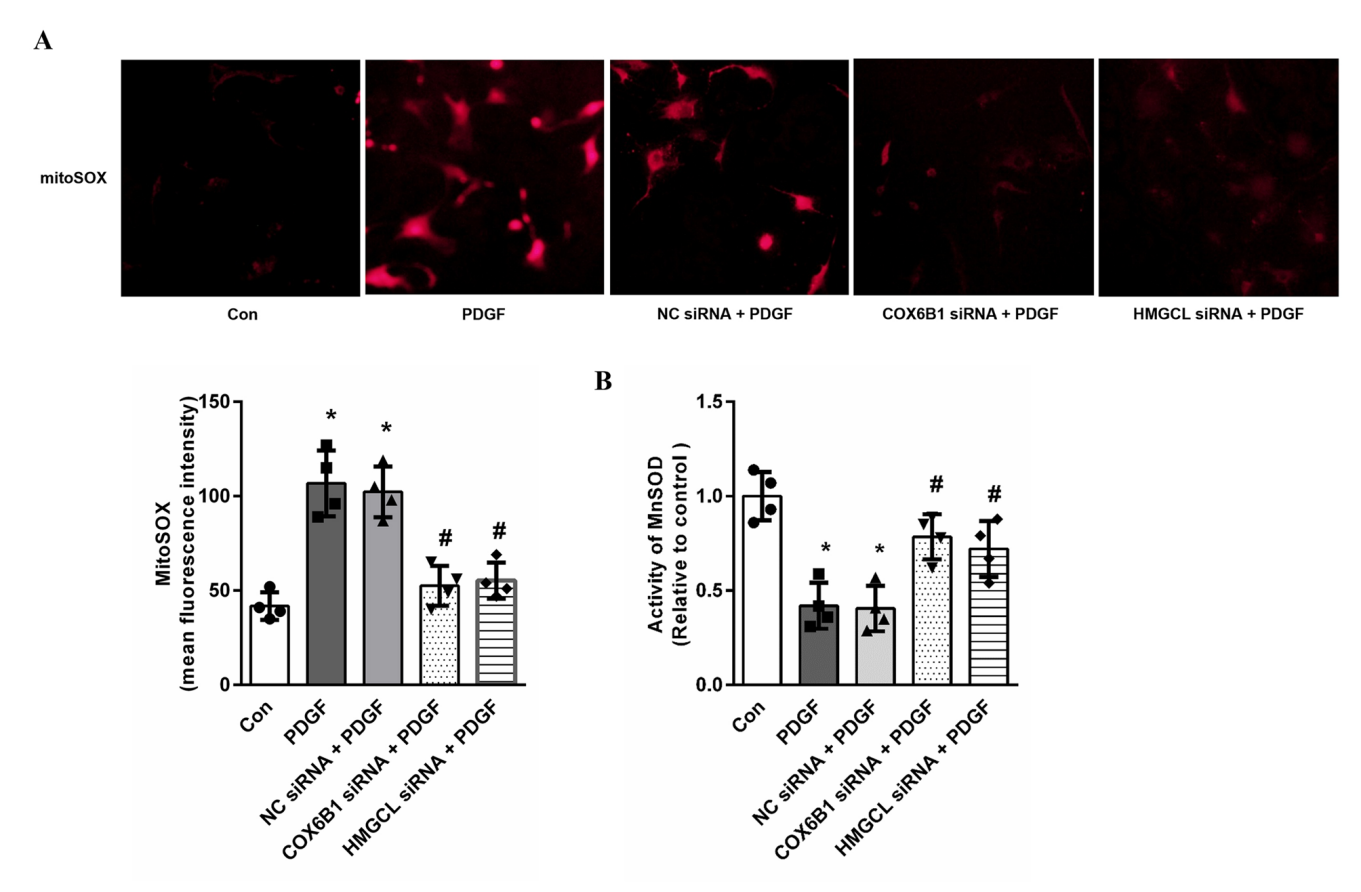
Knockdown of hub genes inhibits mitochondrial oxidative stress in PDGF-induced PASMCs. PASMCs were transfected with non-targeting siRNA or COX6B1 and HMGCL siRNA for 24 h before PDGF stimulation (10 ng/ml) for 24 h, mitoSOX level and activity of MnSOD were examined. (**A**) Representative images of MitoSOX fluorescence and MitoSOX mean fluorescence intensity are shown (*n* = 4 per group). *P < 0.05 versus control group, ^#^*P* < 0.05 versus PDGF-treated cells. (**B**) The activity of MnSOD are shown (*n* = 4 per group). *P < 0.05 versus control group, ^#^*P* < 0.05 versus PDGF-treated cells.

In order to further explore the functional roles of these two hub genes in PDGF-induced PASMCs, we knocked down the COX6B1 and HMGCL in transcriptional level to evaluate if silencing COX6B1 or HMGCL inhibited mitochondrial oxidative stress of PASMCs in PAH. Cells were transfected with COX6B1 or HMGCL specific siRNAs for 24 h and then stimulated with 10 ng/ml PDGF for 24 h, mtROS level and MnSOD activity were determined. MtROS was evaluated using the fluorescent mitochondrial superoxide indicator MitoSOX. As shown in Fig. 8A, mitoSOX fluorescence intensity was significantly higher in PDGF-induced PASMCs compared with control, while knockdown of COX6B1 or HMGCL by siRNA transfection abolished the marked increase in MitoSOX fluorescence intensity induced by PDGF in PASMCs. Figure 8B shows that there is a significant decline in the activity of MnSOD in PDGF-induced PASMCs in comparison to the control group. However, Pre-silencing of COX6B1 or HMGCL by siRNA transfection recover the activity of MnSOD. These results suggest that COX6B1 and HMGCL might involve in mitochondrial oxidative stress in PDGF-induced PASMCs.

## Discussion

PAH is a devastating disorder affecting the pulmonary blood vessels and resulting in irreversible vascular remodeling, which has a major impact on the health and life quality of patients^7^. IPAH, an important subgroup of PAH, occurs independently of other disease processes^8^. The early diagnosis of IPAH presents challenges due to its nonspecific symptomatology, with common manifestations including dyspnea and fatigue. Furthermore, current treatments mainly focus on relief symptoms, rather than directly targeting pulmonary vascular remodeling, leading poor prognosis of IPAH^9^. Prior research has suggested that the occurrence of mitochondrial oxidative stress plays a role in the advancement of PAH^10^. Nevertheless, it has not been reported whether mitochondrial oxidative stress is also involved in the development and progression of IPAH. In this study, we employed bioinformatics analysis and experimental validation to examine the impact of mitochondrial oxidative stress on IPAH.

In this present study, we first identified 3043 DEGs that were differentially expressed in lung tissues from IPAH patients compared to control groups, in order to explore the potential diagnosis biomarker for IPAH. In the WGCNA, a total of eleven gene modules were recognized, in which the blue module were the highly correlated modules with IPAH. To analyze mitochondrial oxidative stress, the study made use of Molecular Signatures Database and MitoCarta3.0 databases to identify genes associated with mitochondrial oxidative stress and utilized the DEGs and WGCNA module to filter out 18 candidate genes for further investigation. Meanwhile, we performed GO and KEGG analysis to reveal the functions of these candidate genes. GO enrichment analysis showed that these genes were engaged in diverse biological processes associated with mitochondrial oxidative stress, including organic acid catabolic process, carboxylic acid catabolic process, amino acid metabolic process, peroxisomal matrix, microbody lumen, ligase activity, and lyase activity. KEGG enrichment analysis indicated potential involvement of these genes in the development of IPAH through pathways related to fatty acid degradation, valine, leucine and isoleucine degradation, tryptophan metabolism. The analysis of functional enrichment suggested that metabolism disorder contributed to the development and progression of IPAH. It has been demonstrated that mitochondrial reactive oxygen species (mtROS) production in cells drives excessive glycolysis, leading to cellular metabolic dysregulation, which exacerbates mtROS generation and triggers a vicious cycle, and ultimately disrupts cellular homeostasis in PAH^11,12^.

Second, we filtered out 2 core hub genes (COX6B1 and HMGCL) by constructing a combination of Lasso regression, SVM-RFE machine learning algorithm and RF algorithm. COX6B1 (cytochrome oxidase subunit 6B1), one of two small subunits in subunit 6 of cytochrome oxidase, mediates the dimerization of COX monomers, and participate in cell respiratory chain transfers^13^. Dysregulation of COX6B1 affect the function of cytochrome oxidase, which potentially mediates the development of some diseases, such as brain myopathy^14,15^. Further studies have shown that COX6B1 significantly reduces the apoptosis of cardiomyocytes and hippocampal neurons after hypoxia/reoxygenation^16,17^. HMGCL (3-hydroxymethyl-3-methylglutaryl-CoA lyase), a metabolic enzyme to catalyze the cleavage of HMG-CoA into acetyl-CoA and acetoacetic acid, involves in the last step of leucine degradation and regulates various biological processes^18^. HMGCL has been found to be markedly upregulated in pancreatic cancer, promoting β-OHB production and providing additional energy for proliferation and metastasis of pancreatic cancer^19^. HMGCL also induces ROS production in nasopharyngeal carcinoma^20^. In this study, COX6B1 and HMGCL showed good ability to distinguish between control and IPAH patients according to the ROC curve analysis. Based on the 2 genes, a diagnostic model was constructed. This model exhibited remarkable diagnostic efficiency values, demonstrating its promising capability for the accurate prediction of IPAH.

Third, the expressions of COX6B1 and HMGCL were validated in MCT-induced PAH animal model, the functional roles of these genes were further explored in PDGF-induced PASMCs. The RT-qPCR and western blot findings showed that both the mRNA and protein levels of COX6B1 and HMGCL were also significantly elevated in MCT-induced rats, consistent with the bioinformatics predictions. Immunohistochemical staining indicated that COX6B1 and HMGCL were predominantly localized in the medial layer of the pulmonary vasculature. Our results further revealed that COX6B1 and HMGCL promoted mtROS production and inhibited MnSOD activity in PDGF-induced PASMCs. These results in our study suggest that upregulation of COX6B1 and HMGCL contributes to mitochondrial oxidative stress in the pathogenesis of IPAH. Study in calorie restriction has found that COX6B1 promotes the formation of supercomplexes, thereby regulating mitochondrial function15. Furthermore, COX6B1 has also been shown to be associated with mitochondrial oxidative phosphorylation and energy metabolism, therefore affecting the progression of tumor^21^. Another study has shown that LncRNA SNHG15 competes with COX6B1 to bind miR-30b-3p through a ceRNA mechanism to affect proliferation, migration, and invasion of lung adenocarcinoma cells^22^. Study has also shown that HMGCL enhances H3K27ac modification by promoting acetyl-CoA production, and upregulates FOXM1 expression, which in turn promotes the nuclear translocation of β-catenin, ultimately contributing to glioma stem cell maintenance and glioblastoma multiforme progression^23^. In view of this, we hypothesize that the role of COX6B1 in the pathogenesis of IPAH might be achieved by affecting mitochondrial oxidative stress, mitochondrial oxidative phosphorylation and energy metabolism; HMGCL might participate in metabolic modulation of histone acetylation in IPAH pathogenesis. However, there is currently a lack of direct evidence to support these notions, which deserves further research to validate.

There are several limitations in this study. Firstly, this bioinformatic analysis was primarily based on a single microarray dataset (GSE15197), which might be influenced by platform-specific biases or cohort-specific characteristics, despite this dataset is well-established and provided a homogeneous cohort for our discovery phase. Future studies integrating multiple datasets from diverse sources and platforms are essential to validate and reinforce our findings. Secondly, the expressions of COX6B1 and HMGCL validation in tissue samples of IPAH patients were lacking, which might affect the interpretation of our results. The exact expression patterns and roles of the identified biomarkers might differ between rodent models and human patients due to species-specific differences. Thirdly, the absence of co-staining with cell-specific markers in our Ki67 and TUNEL assays means that we could not definitively assign the observed proliferation and apoptosis to PASMCs.

## Conclusion

In conclusion, our findings investigated the correlation between mitochondrial oxidative stress and diagnosis of IPAH through bioinformatics analysis, and identified 2 key genes (COX6B1 and HMGCL) might be potential biomarkers for diagnosis of IPAH and potential molecular targets. Further research is needed to validate the specific functions of COX6B1 and HMGCL in mitochondrial oxidative stress and IPAH pathogenesis.

## Materials and methods

### Data sources

Gene expression profiles in GSE15197 dataset of IPAH and control were downloaded from the Gene Expression Omnibus (GEO) database. GSE15197 dataset was deposited by Rajkumar R et al. to identify mutually distinct molecular signatures in IPAH and PH Secondary to idiopathic pulmonary fibrosis (IPF). GSE15197 dataset includes 39 lung tissue specimens from IPAH subjects (*n* = 18), IPF subjects with secondary PH (*n* = 8), and normal controls (*n* = 13) obtained from the University of Pittsburgh Health Sciences Tissue Bank, and from the following GPL6480 platform: Agilent-014850 Whole Human Genome Microarray 6.

### Identification of DEGs

Differentially expressed genes (DEGs) between IPAH patients and normal controls in GSE15197 dataset were screened using limma package in R software. The fold change (FC) in gene expression was calculated. The p-value was adjusted for multiple testing using the Benjamini-Hochberg false discovery rate (FDR) method. |log_2_FC| ≥ 0.85 and FDR-adjusted P-value < 0.05 was defined as criteria for DEGs identification. The ggplot2 and heatmap packages were used to draw the volcano plot and heatmap of DEGs, respectively.

### Weighted gene coexpression network analysis

Weighted gene coexpression network analysis (WGCNA), a widely used algorithm, was performed on GSE15197 dataset to build potential modules between IPAH patients and normal controls. First, a weighted adjacency matrix was constructed and the optimal soft-thresholding power (β) was selected to emphasize strong gene correlations while downweighting weak associations. Gene connectivity within the network was quantified using topological overlap matrix (TOM), which measures the gene interconnectedness based on their shared neighbors. Genes were then clustered hierarchically using average linkage clustering with TOM-based dissimilarity, resulting in distinct co-expression modules represented by color-coded branches in the dendrogram. Furthermore, interactions between gene modules and clinical phenotypes were assessed to identify modules significantly associated with IPAH-related features.

### Identification of candidate mitochondrial oxidative stress-related genes

The expression profiles of oxidative stress-related genes were further extracted from the Molecular Signatures Database. The expression profiles of human mitochondrial genes were extracted from MitoCarta3.0. The intersection genes of DEGs, modules exhibiting the most correlations with IPAH, oxidative stress-related genes and mitochondrial genes were considered to be the candidate genes. Venn diagram was drawn by Venn diagram intersection.

### Functional enrichment analysis of candidate mitochondrial oxidative stress-related genes

Gene Ontology (GO) and Kyoto Encyclopedia of Genes and Genomes (KEGG) pathway enrichment analysis for mitochondrial oxidative stress-related genes were performed by the clusterProfiler package in R software. GO analysis annotates protein functions of the genes in three levels: biological process (BP), cellular component (CC) and molecular function (MF). KEGG analysis integrates the molecular interaction, reaction and relation networks of the genes^24–26^. The result with *P* < 0.05 was considered statistically significant.

### Identification of hub genes

To identify the hub genes potentially involved in the pathogenesis of IPAH from selected mitochondrial oxidative stress-related genes, we employed three machine learning approaches: Least Absolute Shrinkage and Selection Operator (LASSO) logistic regression, Support Vector Machine-Recursive Feature Elimination (SVM-RFE) algorithms and Random Forest (RF) algorithms. LASSO logistic regression was employed to identify parameters showing statistically significant differences between IPAH and control groups by its effectiveness in variable selection and regularization. SVM-RFE algorithm, as a supervised machine learning method, was extensively applied in both classification and regression tasks by its effectiveness in handling high-dimensional spaces and finding the optimal boundary between different classes. RF algorithm gauged the importance of mitochondrial oxidative stress-related genes using the chosen optimal ntree by its effectiveness in handling high-dimensional data and its inherent ability to capture non-linear relationships complemented LASSO and SVM-RFE. Genes that overlapped among the three machine learning algorithms were regarded as hub genes using a Venn diagram tool.

### Diagnostic efficiency of hub genes

A nomogram model based on these hub genes was constructed for the diagnosis of IPAH using multivariate logistic regression. This model assigns weighted point values to each predictor, with “total points” representing their cumulative contribution to risk prediction of IPAH. Furthermore, a receiver operating characteristic (ROC) curve was generated and the area under the ROC curve (AUC) value was calculated to estimate the predictive utility of the hub genes using the pROC package.

### Rat model of PAH

All animal care and experiments were performed in accordance with the Guide for the Care and Use of Laboratory Animals of Xi’an Medical University Animal Experiment Center. All protocols used in this study were approved by the Laboratory Animal Care Committee of Xi’an Medical University. The study was carried out in compliance with the ARRIVE guidelines (http://arriveguidelines.org). The specific details on randomization, blinding, and sample size are provided below. Twelve male Sprague-Dawley (SD) rats were obtained from Animal Experiment Centre of Xi’an Jiaotong University. The rats weighing 180–200 g were kept in the same room with a stable ambient temperature of 22 °C with a 12 h light/dark cycle and housed with free access to a standard diet and tap water. The sample size (*n* = 6 per group) was determined based on previous studies in this field that consistently detected significant effects with similar group sizes, and is consistent with common practice for this model^27,28^. All rats were randomly assigned to either the control group (Con group) or the MCT treatment group (MCT group) using a computer-generated random number sequence prepared by an independent researcher after one week of adaptation. Rats in MCT group received a single intraperitoneal injection of 60 mg/kg of body weight MCT on day 1. Rats in control group received an equal volume of vehicle solution. Due to the visible solution differences, blinding of the investigator during administration was not feasible. However, all subsequent data analysis (histological assessment, biochemical measurements and so on) was performed by an investigator blinded to the group allocation. All survived rats were anesthetized via intraperitoneal injections of 50 mg/kg pentobarbital sodium 28 days after MCT injection. The anesthesia method used in this study was intraperitoneal injection of pentobarbital sodium, which adopts the method of euthanasia for physical interruption of brain activity, in line with the “AVMA Guidelines for the Euthanasia of Animals: 2020 Edition”.

### Measurement of the RVSP and RVH

The right ventricular catheterization was used to determine to mean pulmonary artery pressure (mPAP). RVSP, a well-established surrogate parameter for assessing pulmonary hemodynamics, was assumed to be equal to PAP in the presence of a normal pulmonary valve. Rats were anesthetized and put in the supine position. A polyvinyl catheter filled with heparin-saline solution was inserted from the right external jugular vein into the right ventricle. RVSP was monitored using a Grass polygraph (PowerLab; AD Instruments, Sydney, Australia). The analog signals of pressure were digitized with a sampling frequency of 1,000 Hz and expressed in millimeters of mercury. After hemodynamic measurements, the rats were euthanized via rapid thoracotomy, upon confirmation of deep anesthesia (absence of corneal and pedal reflexes). Death was ensured by exsanguination during this process and confirmed by the absence of a heartbeat. The heart and lungs were immediately excised. The heart was dissected and weighed for calculation of the right ventricular hypertrophy index (RVHI), calculated as right ventricle/left ventricule + septum (RV/LV + S).

### HE staining

The lung morphology was evaluated by HE staining. Right pulmonary lobes were harvested and immersed in 4% paraformaldehyde at room temperature overnight, and then embedded in paraffin wax. Paraffin-embedded tissues were cut to 5 μm in thickness and mounted on glass slides. Tissue sections were stained with hematoxylin and eosin (HE). Pulmonary vascular remodeling was analyzed by measuring the percentage of medial wall thickness (%MT) in vessels (diameter, 50–200 μm), calculated as (2MT/ED) x 100. Vessel external diameter (ED) and medial wall thickness (MT) were measured using a light microscope.

### Immunohistochemistry

To assess the expressions of Ki67, COX6B1 and HMGCL, immunohistochemistry was performed as described. The paraffin-embedded tissue sections were deparaffinized and rehydrated, washed with PBS, incubated in 3% hydrogen peroxide for 10 min, and then blocked with buffered normal goat serum for 30 min at room temperature. Thereafter, the slides were incubated with specific primary antibodies against Ki67, COX6B1 and HMGCL (Proteintech, Wuhan, China; 1:500 dilution) at 4 °C overnight. After being washed with PBS, sections were incubated with horseradish peroxidase-conjugated secondary antibody (Sigma-Aldrich; 1:2000 dilution) at room temperature for 1 h. Finally, the sections were incubated with diaminobenzidine (DAB), dehydrated, coverslipped and observed using a light microscope (Olympus, Japan). The integrated optical density (IOD) was measured using Image Pro Plus 6.0 software.

### TUNEL assay

TUNEL assay was used to detect apoptosis of lung tissue cell using TUNEL kit (Servicebio, Wuhan, China). The paraffin-embedded tissue sections were incubated in xylene to deparaffinized and then rehydrated through a descending ethanol series. Subsequently, tissue sections were incubated in 0.1% Triton X-100 for 8 min on ice, washed with PBS, and then covered with the TUNEL reaction mixture and incubated in a humidified dark chamber (37 °C, 60 min). The lung tissue cells were subsequently counterstained with DAPI (Servicebio) and slices were observed under a microscope.

### Cell isolation and culture

The isolation of primary PASMCs was performed from main pulmonary arteries of male Sprague-Dawley rats weighing 100–150 g. The rats were first anesthetized with an intraperitoneal injection of 50 mg/kg sodium pentobarbital and subsequently sterilized by immersion in 75% alcohol for 3 min. The heart and lungs were excised and rinsed with ice-cold PBS. The main pulmonary arteries were then dissected, cleaned of adherent tissues in ice-cold PBS, and the smooth muscle layer was isolated by scraping off the adventitia and intima under aseptic conditions. The resulting tissue was minced into small pieces (0.5–1 mm²) and placed in a culture flask.

The PASMCs were maintained in a complete culture medium based on Dulbecco’s Modified Eagle Medium (DMEM)/High Glucose (Gibco) containing 10% fetal bovine serum (FBS, Sijiqing, HangZhou, China), 100 U/ml penicillin and 100 µg/ml streptomycin, under standard conditions (37 °C, 5% CO₂). Subculturing was performed with 0.25% trypsin (Invitrogen) when confluence was reached, typically after two weeks. Cells from passages 4–8 were utilized in all studies. Before experimentation, a period of overnight serum deprivation was carried out using medium with 1% FBS. Stimulation of proliferation was induced by PDGF (Novoprotein, Beijing, China), which was prepared as an aqueous stock solution at 1000 µg/mL.

### Small interfering RNA transfection

Gene silencing of COX6B1 and HMGCL was achieved by transfection of PASMCs with either target-specific or non-targeting control siRNA (GenePharm, Shanghai, China), employing Lipofectamine™ 2000 (Invitrogen) as the transfection reagent following the manufacturer’s instructions. In this procedure, primary PASMCs were first seeded into 6-well plates at a density of 5 × 10⁵ cells/well. The siRNA and transfection reagent were individually diluted in serum-free DMEM and allowed to equilibrate separately for 5 min at room temperature. The two solutions were subsequently mixed and incubated for 20 min to allow for complex formation. Transfection was carried out by exposing cells at 30–50% confluence to the siRNA-lipid complexes for 6 h in serum-free medium. Finally, the transfection medium was replaced with complete growth medium, and the cells were maintained for 48 h at 37 °C in a 5% CO₂ incubator.

### Quantitative real-time polymerase chain reaction

The relative mRNA expression levels of hub genes (COX6B1 and HMGCL) were quantified using quantitative real-time polymerase chain reaction (RT-qPCR). Total RNA was isolated from lung tissues using the RNeasy Fibrous Tissue Mini Kit (QIAGEN, Hilden, Germany). The total RNA concentration and purity at the absorbance ratio 260/280 nm was determined using NanoDrop 2000 (Thermo scientific, Waltham, MA, USA). Subsequently, cDNA was synthesized from total RNA using Servicebio^®^RT First Strand cDNA Synthesis Kit (Servicebio) with specific primers. qPCR was performed using SYBR Green qPCR Master Mix (Servicebio, Wuhan, China) on a CFX RT-PCR detection system (Bio-Rad). PCR amplification was performed under the following conditions: 95 °C for 30 s, followed by 40 cycles of 95 °C for 10 s and 60 °C for 30 s. Relative mRNA expression levels were calculated using the 2^−ΔΔCt method, with glyceraldehyde 3-phosphate dehydrogenase (GAPDH) as the internal reference gene for normalization.

### Western blot

Western blot was performed for protein analysis. Lung tissues were homogenized in RIPA buffer supplemented with protease inhibitor and PMSF with a tissue homogenizer. The Tissue lysates were centrifuged at 10,000 rpm for 20 min at 4 °C, and the supernatant was collected as total protein and measured for protein quantification using a BCA assay kit (Pierce, Rockford, IL, USA). The protein samples were mixed with 5× SDS loading buffer and denatured by boiling at 100 °C for 10 min, then separated by 10% sodium dodecyl sulfate-polyacrylamide gel electrophoresis (SDS-PAGE) and subsequently transferred onto polyvinylidene difluoride (PVDF) membranes (Millipore, USA) in a Bio-Rad Trans-Blot system. After blocking with 5% non-fat milk, the membranes were incubated overnight at 4 °C with primary antibodies (COX6B1, HMGCL and GAPDH) (Proteintech, 1:500 dilution). Following washing and incubation with HRP-conjugated secondary antibodies (1:5000 dilution) at room temperature for 1 h, protein bands were detected using an ECL system (Amersham Bioscience) and visualized using the SuperSignal Chemiluminescent Substrate (Invitrogen, Thermo Fisher Scientific).

### Activity of MnSOD

MnSOD activity was assayed using the method as described previously. 32 The reaction mixture consisted of 0.1 M phosphate buffer (pH 7.8), 12 mM L-methionine, 70 µM nitroblue tetrazolium (NBT), 0.2 mM riboflavin and 3 µM EDTA. The reaction was initiated with the addition of the mitochondrial extract obtained after 3 − 4 freeze‐thaw cycles for releasing the matrix content. One set of tubes were illuminated under light for 30 min and another set was kept in the dark. A similar reaction mixture without mitochondrial extract was run simultaneously which was used as control. O.D. was recorded at 560 nm. The difference between light and dark sets was used to calculate unit of SOD. One unit was defined as the amount of enzyme that produced 50% inhibition of NBT reduction/min and activity was calculated as: SOD(U) = ([(C − E)/(C/2) × (total volume of assay mixture/amount of enzyme extract)] Where, C = difference in absorbance of the control tubes kept for incubation in light and dark, E = difference in absorbance of the tubes kept for incubation in light and dark.

The activity of MnSOD was determined using a Cu/ZnSOD and Mn-SOD Assay Kit with WST-8 (Beyotime, Beijing, China) according to the manufacturer’s instructions. Cells were lysed and the supernatant was collected and mixed with WST-8 enzyme working solution in a 96-well plate. The reaction was initiated by adding the enzyme reagent, and the plate was incubated at 37 °C for 30 min. The absorbance at 450 nm was measured using a microplate reader (Bio-Rad, Richmond, CA, USA).

### Mitochondrial reactive oxygen species measurement

Mitochondrial reactive oxygen species was measured using MitoSOX (MedChemExpress, Shanghai, China). Briefly, PASMCs were incubated with MitoSOX working solution (5 µM) for 30 min at 37 °C in the dark followed by 3 washes with PBS. The fluorescence intensities were analyzed by fluorescence microscopy (Olympus Corp.).

### Statistical analysis

All statistical analyses were performed using the R software and GraphPad Prism software. Continuous variables are expressed as mean ± SD. Student’s t-test was used for comparisons of differences between two groups. The Wilcoxon test identifies the significance of any differences between IPAH and control groups. *P* < 0.05 was considered statistically significant.

## Supplementary Information

Below is the link to the electronic supplementary material.

Supplementary Material 1

## Data Availability

Datasets (GSE15197) used in the study can be downloaded from the public GEO database (https://www.ncbi.nlm.nih.gov/geo/query/acc.cgi?acc=GSE15197). The original contributions presented in the study are included in the article. Further inquiries can be directed to the corresponding author.
