# Supplementary figures and images for: Identification of potential biomarkers associated with mitochondrial oxidative stress in idiopathic pulmonary arterial hypertension via bioinformatic and experimental analysis
Source: Sci Rep. 2025 Dec 9;16:1647. doi: 10.1038/s41598-025-31150-7 (PMC12800125; doi:10.1038/s41598-025-31150-7)

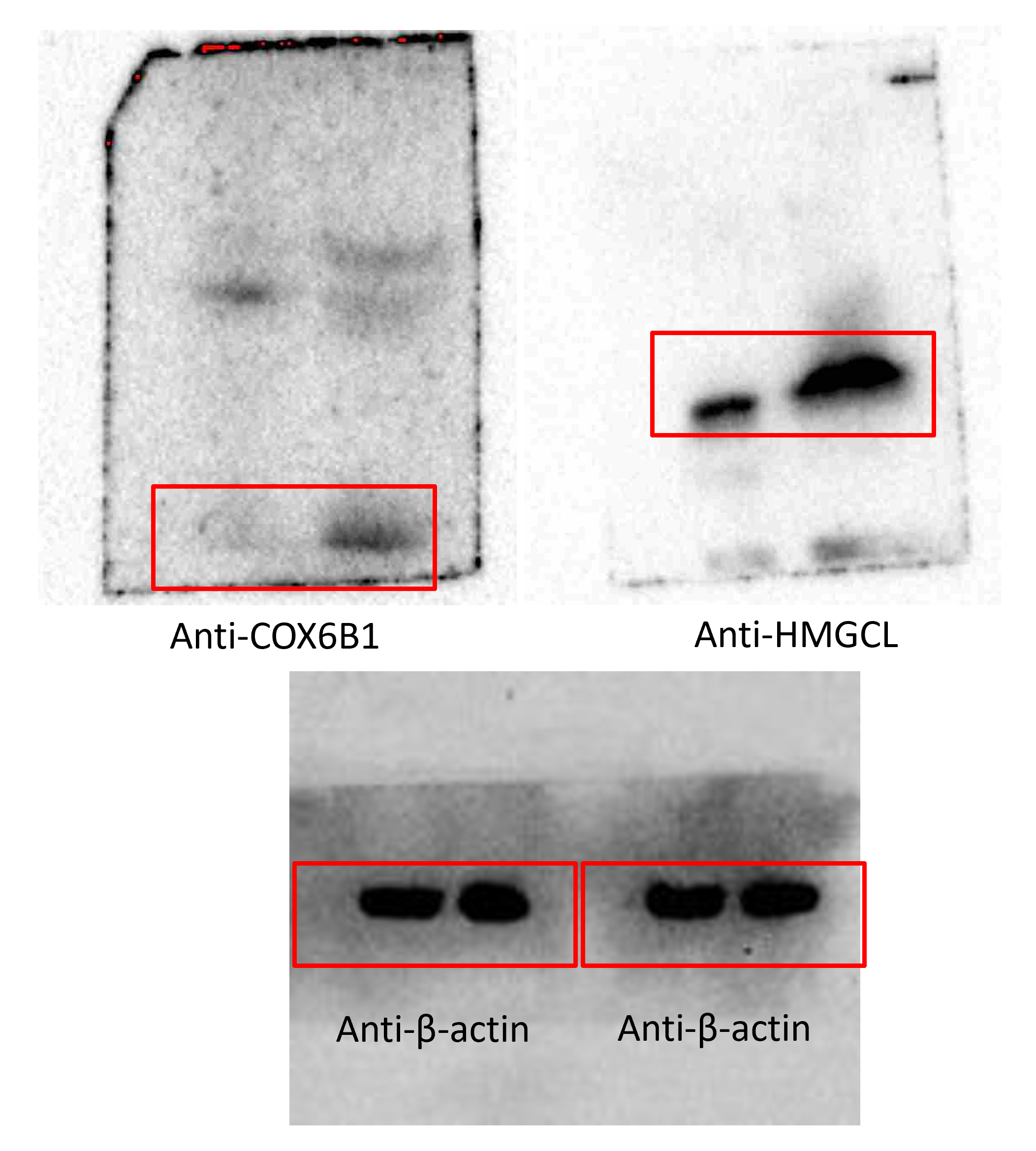

Supplement: Supplementary file 1 — Supplementary Material 1 [file 41598_2025_31150_MOESM1_ESM.png]
